# Effect of 24-form simplified Tai Chi on executive inhibitory control of college students: a randomized controlled trial of EEG

**DOI:** 10.3389/fpsyg.2024.1344989

**Published:** 2024-03-07

**Authors:** Min Wang, Bei Lyu

**Affiliations:** ^1^Public Sports Department, Institute of Physical Education, Huzhou University, Huzhou, Zhejiang, China; ^2^Chinese Graduate School, Panyapiwat Institute of Management, Nonthaburi, Thailand

**Keywords:** inconsistent task, reaction time, accuracy, α band power, β band power, θ band power

## Abstract

**Background:**

College students, undergoing crucial cognitive development, face challenges during the COVID-19 pandemic that impact their executive functions. While existing research indicates positive effects of Tai Chi (TC) on college students’ cognitive abilities, there is a scarcity of studies investigating its impact on executive functions and frontal brain activity.

**Objective:**

This study aimed to compare the effects of 24-form simplified TC training on college students’ executive functions and frontal brain electrical activity. The hypothesis posited that the TC group would exhibit superior performance compared to the control group during COVID-19 pandemic.

**Method:**

Seventy college students were randomly assigned to either TC group or control group, engaging in 36 sessions (3 sessions per week, 45 min each) over 12 weeks. Executive inhibitory control was assessed using the Stroop Color and Word Test, and resting brain electrical activity in the frontal area was recorded through Electroencephalography.

**Result:**

ACC was influenced by group, group-time interaction, and Stroop task-time interaction. RT was affected by time, task condition, task condition-time interaction, and task condition-group interaction. Notably, the TC group showed improved ACC (from 96.54 ± 3.27% to 98.90 ± 1.32%) and decreased RT (from 0.73 ± 0.12 to 0.66 ± 0.07 s), particularly in the inconsistent task. Regarding EEG band power, significant Group and Time interaction effects were found in F3-θ, F3-α, F3-β, F4-θ, and F4-α. Moreover, within the TC group, significant increases in F3-θ band power (from 4.66 ± 3.55 to 7.71 ± 8.44) and F4-θ band power (from 4.41 ± 2.82 to 8.61 ± 9.51) (10^−3^·μV·Hz) were noted pre-and post-tests. In the control group, significant decreases were observed in F3-α band power (from 5.18 ± 4.61 to 2.79 ± 2.11) and F4-α band power (from 5.57 ± 6.58 to 2.48 ± 1.95) (10^−3^·μV·Hz).

**Conclusion:**

The pandemic-induced panic may impact frontal lobe brain activity in college students. TC training not only improves executive inhibitory control but may also enhance localized brain activity, suggesting its potential as a holistic intervention for cognitive and neurological well-being during stressful periods.

## 1 Introduction

College students, importantly integral to society, confront unparalleled mental health obstacles, especially in China, where roughly 20% of students experience diverse psychological issues (Liu et al., 2019). The COVID-19 pandemic has worsened their emotional distress and affected their mental well-being and cognitive abilities. A Spanish study revealed a link between anxiety levels and alterations in executive functions among adolescents during the pandemic (Lavigne-Cerván et al., 2021). Indeed, it is the case that stress and anxiety primarily affect the variability of performance behavior, that is they are energetic aspects of cognitive control, which are expressed in metrics other than the mean reaction time (Schumann et al., 2022). Tai Chi (TC) exercise can improve in attention, memory, executive control, and stress reduction. By integrating physical movement with mindfulness practices, TC exercise has positive effects on precisely these energetic aspects of behavior and performance.

Specifically, TC exercise engages executive control processes by requiring practitioners to plan and execute complex movement sequences, maintain balance and posture, and adapt to changes in environmental conditions. These processes include inhibitory control, working memory, and cognitive flexibility. Energetic constraints may affect executive control by limiting the cognitive resources available for inhibitory control, working memory maintenance, and task switching. For example, maintaining balance and executing precise movements during TC practice requires sustained attention and motor control, which may be compromised under conditions of physical fatigue or cognitive overload. Additionally, stress or anxiety can deplete cognitive resources needed for executive control, leading to decreased performance and increased susceptibility to errors. Exactly, TC has a basic ability of “energetic regulation” to attain and maintain a state of general optimal activation for upcoming demands, to set an optimal rhythm, and to maintain this rhythm for the demand.

Executive functions, indicative of advanced cognitive capabilities, often involving an interconnected network of brain regions, like as PFC networks, play critical roles (Levit et al., 2020). Previous studies have explored the effects of various exercises on brain function. Jiang et al. (2019) reported increased alpha and beta wave power values after limb movement training, while (Gutmann et al., 2015) found no change in individual alpha peak frequency after moderate exercise. Additionally, it reported that intense exercise increases beta oscillation capacity in frontal and central regions of the brain, which may indicate increased cortical activation (Hübner et al., 2018), however, executive functions are distinguished from other functions, for example, Posner et al. argued for viewing attention function as (1) alerting, (2) orienting, (3) selecting, (4) and executive attention, with executive functions specifically dealing with resolving conflicts in behavioral tendencies (Posner, 2016), and the Stroop Color-Word Task in this study primarily assesses inhibitory control (Miyake and Friedman, 2012).

TC, known for deliberate movements, synchronized breathing, and mindfulness, regard as an optimal choice for both physical exercise and the development of attention and mindfulness (Di Liegro et al., 2019). Empirical evidence supports the positive effects of TC on attention deficit (Converse et al., 2020) and fluid intelligence (Lv et al., 2022) in healthy college students. It has also demonstrated potential in averting cognitive decline (Tao et al., 2017; Sun and Lyu, 2022), aiding dementia (Sungkarat et al., 2017), facilitating stroke recovery (Taylor-Piliae et al., 2011), and enhancing strength, balance, memory, and attention (Nguyen and Kruse, 2012), highlighting the need for further exploration in inhibitory control, particularly regarding the long-term effects of TC training (Parvin et al., 2020).

Electroencephalography (EEG) measures the electrical activity of the brain by recording and analyzing the electrical signals generated by neurons (Celia et al., 2022). Several types of brainwaves can be observed using EEG. These brainwaves are categorized based on their frequency, which is measured in Hertz, including alpha, beta, theta, or gamma, and they each correspond to different states of consciousness, mental activity, and physiological processes. When say that certain activities have effects on alpha, beta, theta, or gamma activity using EEG, it refers to how those activities influence the strength, frequency, or synchronization of these specific bands of brainwaves. For example: Meditation or relaxation techniques may increase alpha activity, leading to a state of calmness and relaxation. Engaging in cognitive tasks or problem-solving activities may increase beta activity, reflecting heightened mental alertness and focus. Deep sleep or unconsciousness is associated with increased delta activity, indicating a state of deep relaxation and restorative sleep. Creativity or daydreaming may be accompanied by increased theta activity, reflecting a relaxed and imaginative state. When comparing rs-EEG data between different activity groups in the context of executive inhibitory control, different bands of brainwave power in potential differences would be interesting. If the TC group exhibits higher theta or beta power/synchronization compared to the control group during the resting state, it could suggest that TC practice enhances the overall readiness or efficiency of inhibitory control networks in the brain. Conversely, if the control group shows higher power/synchronization in these frequency bands, it could indicate that TC practice does not significantly affect resting-state inhibitory control mechanisms measured by rs-EEG. Functional magnetic resonance imaging (fMRI) has documented that TC practice can impact the structure and functionality of the frontal lobes of the brain (Wei et al., 2014), augmenting memory (Tao et al., 2016). Employing similar methodologies, researchers have identified increased cortical thickness in the frontal and occipital regions among TC practitioners (Pan et al., 2018), while electroencephalogram (EEG) data highlights significant θ activity in the frontal-central and central-occipital cortical areas (Liu et al., 2003). TC has also been substantiated in reducing anxiety and depression (Liu et al., 2023). Despite these observations (Xie et al., 2019), the specific mechanisms through which TC influences brain function during movement remain largely unexplored.

This study focuses on the impact of the 24-form simplified TC, also known as the Simplified TC, which is a popular sequence of TC movements created in 1956 by the Chinese National Physical Education Committee. It emphasizes slow, deliberate movements coordinated with deep breathing, promoting a sense of calm and inner peace. Compared to longer traditional TC forms, the 24-form is shorter and easier to learn, making it a popular choice for beginners and those with limited time for practice. On the executive inhibitory control of college students, utilizing a randomized controlled experimental design to offer empirical validation and understand the regulatory effects on frontal lobe activity. We hypothesized that TC practice could improve executive inhibitory control capabilities and increased the alpha, beta, and theta band power of the prefrontal lobe of the brain.

## 2 Material methods

### 2.1 Participants

This study employed a randomized controlled trial design. Prior to the commencement of the experiments, participants were provided written information detailing the experimental procedures, and their consent to participate was obtained. Subsequently, interested participants completed questionnaires to ascertain their eligibility. Eligible participants were then randomly allocated to either the TC group or the control group. Before the intervention, all participants underwent a battery of cognitive and physical fitness assessments to evaluate the impact of the TC intervention on their executive inhibitory control.

Participants were recruited from Huzhou University in China. All participants were 18 to 22 years old. The participants were screened using the following inclusion criteria: (1) could be independently ambulant and had an absence of musculoskeletal pain or condition that limits exercise practice, (2) were able to participate in a low-moderate intensity gentle movement group for 12 consecutive weeks, (3) had a normal or corrected vision, and no color blindness, (4) they are neither smoker nor do they drink too much alcohol, (5) had not taken antioxidant supplementation.

The exclusion criteria were: (1) regular participated in any type of professional medication exercises, such as yoga, or jogging, (2) reporting any history or risk for cardiopulmonary disease, (3) being pregnant or suspected to be pregnant, (4) experienced in applying cognitive tasks or any limitations in the ability to complete cognitive testing procedures within in 3 years, and (5) consumed any caffeinated or alcoholic products for 24 h before their experimental session.

All participants provided written informed consent before enrolling in the study. To present the study design, a sample size of 70 participants for the interaction measurement groups was calculated to be appropriate. Considering about a 30% attrition rate due to COVID-19 pandemic, we recruited 98 total participants. Finally, 35 participants were included in the TC group and 35 in the control group.

### 2.2 Study design

This study protocol was approved by the Center for Ethics in Human Research, Khon Kaen University, and Recorded No.4.2.01: 6/2565 (Reference No. HE652012) and registration number ChiCTR2200059427. This was a single-blind (assessor), parallel, randomized controlled trial. Seventy college students were randomly assigned to either the TC group or the control group via a sealed envelope within a 2 (TC group vs. control group) × 2 (pre-test vs. post-test) factorial design. Pre-and post-intervention executive function assessments and brain rs-EEG studies, described in the Outcomes’ measures section below, were performed by another blinded assessor in the team.

### 2.3 Intervention

The TC training of the TC group will begin in April 2022, three times a week for 12 consecutive weeks, 2 of which will be supervised by 2 experienced TC coaches in a group format. Each training lasts about 45 min, including 5 min of warm-up, 35 min of practice, and 5 min of relaxation, in addition, the team members also did not participate in other regular exercises. Participants’ session attendance was recorded by the researcher, and the compliance rate was 100%, and the team members were required to wear Xiaomi sports bracelets to monitor about 55% of the maximum heart rate (Pang et al., 2021).

The participants in the control group remained devoted to maintaining their daily routines and refrained from any new exercise interventions for 12 weeks.

### 2.4 Data collection

The evaluation of outcomes took place at two points in time: baseline, conducted 1 day before the TC intervention, and post-intervention, conducted 1 day after the completion of the 12-week intervention. All data were gathered through computerized methods or instruments.

#### 2.4.1 Anthropometry

To ensure the reliability and precision of anthropometric data collection, the study implemented standardized procedures. Participants, without shoes, underwent height measurement using a height scale (Jiangsu Suhong Medical Equipment Co., Ltd., China) and weight measurement using the Huawei Smart Scale 3 Pro (Huawei, China). Each participant stepped on the respective height and weight scales twice for approximately 15 s. Body Mass Index (BMI) was calculated using the weight/height^2^ ratio.

#### 2.4.2 Executive function

Executive function encompasses various cognitive dimensions, including inhibitory control, decision-making, working memory, and reward sensitivity, collectively facilitating planning, problem-solving, flexible reasoning, and behavioral and emotional regulation (Ronan et al., 2020). The Stroop Color-Word Task (SCWT) was employed to assess executive function, measuring accuracy and reaction time during congruent and incongruent trials.

The SCWT, a widely used neuropsychological test for evaluating cognitive control and the ability to inhibit automatic responses (Ktaiche et al., 2022), was programmed using E-prime 2.0 (Psychology Software Tools, Inc., Pittsburgh, PA).

**Figure 1:**
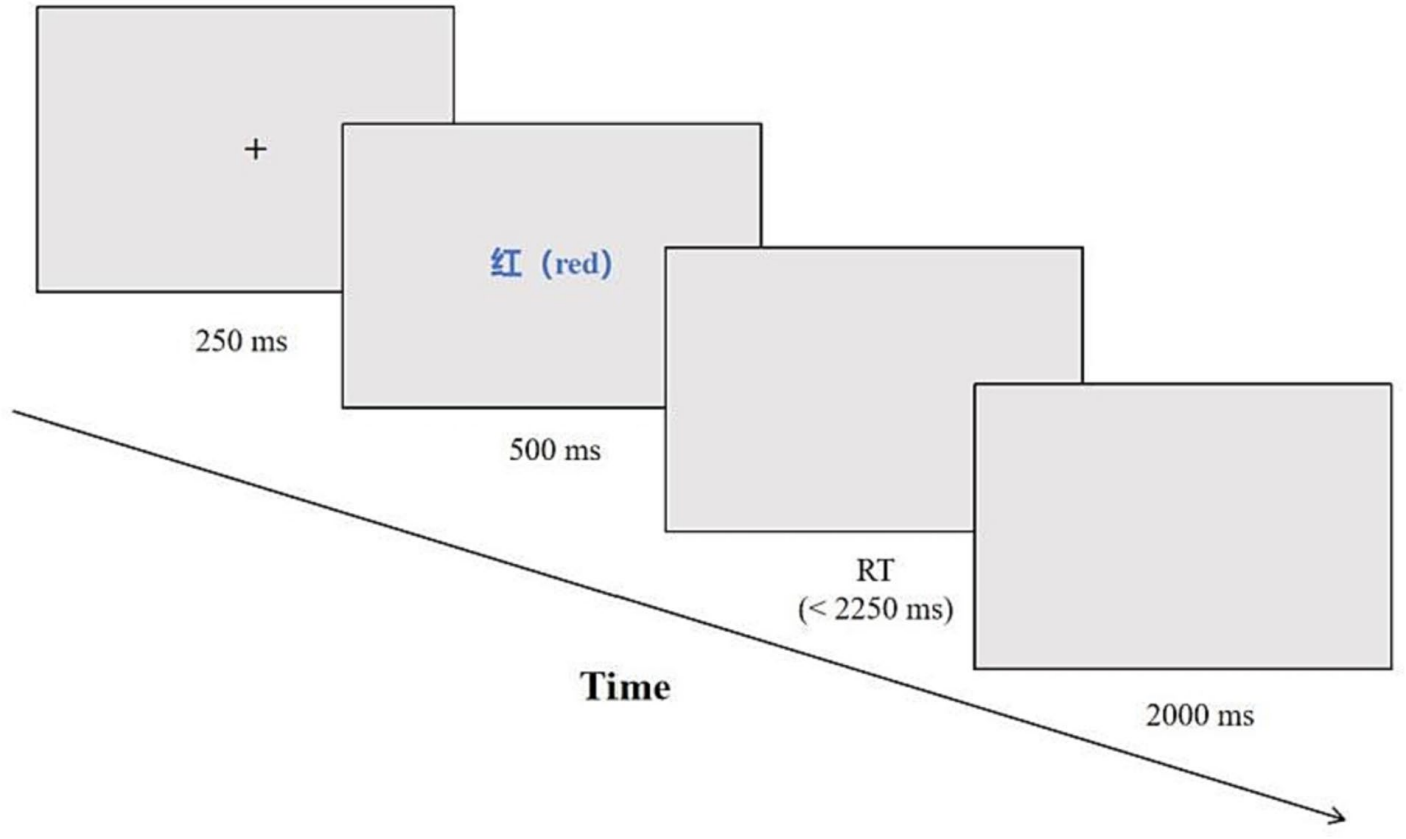
The flow diagram of Stroop test.

All visual stimuli were centrally presented on the display screen, featuring a white background and characters displayed in Microsoft Yahei font at 48-point size, bold, with an opaque black style. Participants were instructed to respond by pressing one of four keys (f, j, r, or u) corresponding to the color of the Chinese text presented—red, yellow, blue, or green, respectively. The correct response was determined by the finger assigned to the corresponding color, such as pressing the “f” key with the left index finger when the Chinese text appeared in red. The stimuli comprised randomly presented combinations of colors and Chinese text, including 120 incongruent and 40 congruent trials. The total duration of all stimuli was 3,000 ms, with a fixation point (+) displayed for 250 ms, followed by the presentation of a visual stimulus lasting 500 ms. This was followed by a black screen that lasted for a maximum of 2,250 ms, or until the participant pressed a key. The inter-trial interval was 2000 ms, which was used to separate different trials, as shown in Figure 1. In this study, participants’ correct response times (RT) were recorded, and errors were handled, including the accuracy (ACC) of post-error trials.

To assess the conflict effect before and after TC training (Xu et al., 2021), RT and ACC rates were utilized. Participants were required to quickly respond to “color” stimuli while accurately suppressing responses to “Chinese text” stimuli, necessitating inhibitory control to overcome automatic response tendencies. RT was measured from the appearance of the stimulus to the time when the subject pressed the button, and response ACC referred to the accuracy of the subject’s response in both consistent and inconsistent stimulus conditions. A shorter RT or higher response accuracy, or a combination of both, indicated stronger inhibitory control. Prior to the formal trial, all participants underwent practice sessions until their accuracy exceeded 80%.

#### 2.4.3 Brain oscillation

Electroencephalography (EEG; Brain Products GmbH, Munich, Germany) was utilized to study the brain’s electrical activity during rest, providing insights into functional connectivity between brain regions associated with various mental states, such as sleep, meditation, and cognitive processing.

Participants wore a 64-channel EEG cap (Brainamp MR Plus, Brain Products GmbH), and EEG data were recorded with electrodes at F3 and F4 positions according to the International 10–20 System. Ground and reference electrodes were positioned on the forehead and the tip of the nose, respectively (Wiens et al., 2019).

EEG recordings were conducted in an awake state with closed eyes for 8 min, and power spectrum densities in the α (8–12 Hz), β (13–30 Hz), and θ (4–8 Hz) frequency bands in the frontal lobes were assessed before and after TC practice. The data were sampled at 1000 Hz and an impedance of less than 50 kΩ at all recording electrodes (Kang et al., 2019).

Brain Vision Analyzer (Version 2.2.0; Brain Products GmbH, Munich) was used for EEG data preprocessing. The data were re-referenced to the average reference, a notch filter (50 Hz) was applied to remove power-line noise, and IIR filters were applied for low-pass (cut off 40 Hz) and high-pass (cut off 0.5 Hz) filtering. The semiautomatic inspection identified and removed artifacts, and the remaining data were subjected to Fast Fourier Transform (FFT), and the duration of 2-s artifact-free epochs (Nash et al., 2022) was exported for power spectrum density analysis. The resulting values were averaged for each participant to quantify resting-state EEG power.

### 2.5 Statistical analyses

All statistical analyses were performed using IBM SPSS Statistics version 26.0 (IBM Corp., Armonk, NY). The Shapiro–Wilk normality test first determined that the data obeyed the normal distribution, and data are reported as mean and standard deviation. According to the study’s design, two-way repeated ANOVA was used to analyze data. For Stroop performance measurement, it contains the between-subject factor group (2 levels: tai-chi group vs. control group), the within-subject factor session (2 levels: pretest vs. posttest), and the within-subject factor Stroop congruence (compatible vs. incompatible). An alpha level of 0.05 was used for all statistical tests.

## 3 Results

### 3.1 Study overview and participant characteristics

Throughout the 12-week TC training, participants reported no adverse events, demonstrating the safety and well-tolerance of the intervention. Baseline characteristics, except for age, did not significant difference between the TC and control groups, see Table 1.

**Table 1:** Demographic characteristics of participants (*n* = 45).

Variables	Tai Chi group M ± SD	Control group M ± SD	*p*-value
Demographic			
Age	20.08 ± 0.28	20.71 ± 0.51	0.01**
Gender (male/female)	10,25	6,29	0.26
BMI (kg/m^2^)	22.24 ± 3.07	21.30 ± 4.00	0.28
Cognitive performance			
incongruent ACC (%)	96.54 ± 3.27	96.76 ± 3.56	0.79
incongruent RT (s)	0.73 ± 0.12	0.77 ± 0.13	0.25
congruent ACC (%)	97.57 ± 3.10	97.42 ± 2.53	0.83
congruent RT (s)	0.67 ± 0.12	0.68 ± 0.13	0.63
EEG (10^−3^·μV·Hz)			
F3-θ	4.66 ± 3.55	5.70 ± 5.70	0.37
F3-α	5.45 ± 5.95	5.18 ± 4.61	0.83
F3-β	0.89 ± 0.60	1.00 ± 0.94	0.56
F4-θ	4.41 ± 2.82	5.12 ± 5.72	0.51
F4-α	4.88 ± 5.51	5.57 ± 6.58	0.64
F4-β	0.90 ± 0.56	0.87 ± 0.78	0.82

BMI, Body Mass Index; ACC, accuracy; RT, reaction time. Use * for *p* < 0.05 and ** for *p* < 0.01.

### 3.2 Changes in executive inhibitory control after 12 weeks of TC intervention

It showed that Accuracy (ACC) had a significant effect in accordance with the group effect (*p* = 0.05), the interaction effect of “group and time” (*p* = 0.04), and the interaction effect of “the Stroop task condition and time” (*p* = 0.01). Additionally, within the TC group, there was a significant improvement in ACC for the inconsistent task (from 96.54 ± 3.27% to 98.90 ± 1.32%), with a notable disparity between pre-and post-tests. Furthermore, in the post-test, a significant divergence in ACC was observed between the TC and control groups for both inconsistent and consistent tasks.

Reaction time (RT) had a significant effect in accordance with the time effect (*p* = 0.02) and the Stroop task conditions effect (*p* < 0.01). Moreover, Reaction time also had the interaction effect of “the Stroop task conditions and group” (*p* = 0.01) and “the Stroop task condition and time” (p < 0.01). Furthermore, within the TC group, a significant decrease in RT for the inconsistent task (from 0.73 ± 0.12 to 0.66 ± 0.07 s) was observed between pre-and post-tests, and there was a significant difference between the TC group and the control group in the post-test RT of the incongruent task. Additionally, there were significant differences between RTs across the Stroop task conditions, see Table 2.

**Table 2:** Effects on executive inhibitory control after 12 weeks of TC intervention.

Variables		Tai Chi Group M ± SD		Control Group M ± SD		*p*-value						
		Pre-test	Post-test	Pre-test	Post-test	Group	Time	Group ×Time	Stroop task condition (STC)	STC× Group	STC× Time	STC × Group × Time
ACC (%)	incongruent	96.54 ± 3.27	98.90 ± 1.32^a,b^	96.76 ± 3.56	96.98 ± 3.50	0.05	0.25	0.04	0.74	0.94	0.01	0.49
	congruent	97.57 ± 3.10^c^	98.07 ± 2.58^b^	97.42 ± 2.53	96.42 ± 3.39							
RT (s)	incongruent	0.73 ± 0.12	0.66 ± 0.07^a,b^	0.77 ± 0.13	0.71 ± 0.10	0.06	0.02	0.65	<0.01	0.01	<0.01	0.92
	congruent	0.67 ± 0.12^c^	0.64 ± 0.06^c^	0.68 ± 0.13^c^	0.67 ± 0.09^c^							

STC, Stroop task condition; ACC, accuracy; RT, reaction time. ^a^Represents the significance before and after within the group; ^b^Represents the significance between the Tai Chi group and the control group in the post-test; ^c^Represents the significance between the inconsistent task and the consistent task.

### 3.3 Changes in EEG after 12 weeks of TC intervention

There was a significant time effect in F4-θ band power (*p* = 0.05), and F3-θ, F3-α, F3-β, F4-θ, and F4-α band power exhibited significant Group and Time interaction effect (all *p* < 0.05). Secondly, within the TC group, significant differences were noted in F3-θ band power (from 4.66 ± 3.55 to 7.71 ± 8.44) and F4-θ band power (from 4.41 ± 2.82 to 8.61 ± 9.51) (10^−3^·μV·Hz) between pre-and post-tests. Simultaneously, significant differences in F3 and F4-θ band power were observed in the post-test between the TC group and the control group (Table 3).

**Table 3:** Effects on brain performance after 12 weeks of TC intervention.

Variables (10^−3^·μV·Hz)	Tai Chi Group M ± SD		Control Group M ± SD		*p* value		
	Pre-test	Post-test	Pre-test	Post-test	Group	Time	Group × Time
F3-θ	4.66 ± 3.55	7.71 ± 8.44^a,b^	5.70 ± 5.70	3.75 ± 2.05	0.19	0.45	0.01
F3-α	5.45 ± 5.95	5.41 ± 6.31^b^	5.18 ± 4.61	2.79 ± 2.11^a^	0.17	0.05	0.05
F3-β	0.89 ± 0.60	1.06 ± 1.78	1.00 ± 0.94	0.58 ± 0.38^a^	0.39	0.41	0.04
F4-θ	4.41 ± 2.82	8.61 ± 9.51^a,b^	5.12 ± 5.72	3.63 ± 2.13	0.07	0.07	<0.01
F4-α	4.88 ± 5.51	6.18 ± 6.88^b^	5.57 ± 6.58	2.48 ± 1.95^a^	0.20	0.18	0.01
F4-β	0.90 ± 0.56	0.97 ± 1.31	0.87 ± 0.78	0.57 ± 0.33	0.19	0.29	0.09

^a^Represents the significance before and after within the group; ^b^Represents the significance between the Tai Chi group and the control group in the post-test.

In the control group, significant differences were found in F3-α band power (from 5.18 ± 4.61 to 2.79 ± 2.11) and F4-α band power (from 5.57 ± 6.58 to 2.48 ± 1.95) (10^−3^·μV·Hz), as well as F3-β band power (from 1.00 ± 0.94 to 0.58 ± 0.38) (10^−3^·μV·Hz), between the pre-and post-tests. Moreover, a significant difference was observed in the F3-α band power of the inconsistent task post-test between the TC group and the control group.

## 4 Discussion

As our comprehension of the intricate connection between the body and mind advances, TC, as a mind–body practice, is gaining increasing attention from the research community. This study reveals that the TC group exhibited enhanced executive inhibitory control, accompanied by an overall elevation in frontal lobe activation. This offers a point to explore the regulation of frontal lobe activity by TC and its potential repercussions on cognitive functions.

It’s well-documented that heightened levels of anxiety and stress, as experienced during the COVID-19 pandemic, can detrimentally affect executive inhibitory control (Clay et al., 2023). Chronic stress is often associated with structural and functional alterations in critical brain regions like the prefrontal cortex and anterior cingulate cortex, pivotal for executive function and inhibitory control. Functional neuroimaging studies have delineated changes in brain activation patterns during tasks requiring inhibitory control among individuals grappling with heightened anxiety or stress levels, suggesting a plausible association between COVID-19-induced panic and alterations in neural processing pertinent to executive function. Moreover, COVID-19 panic might exert influence on cognitive performance via attentional biases and rumination, impeding effective regulation of thoughts and behaviors. Individuals under the grip of COVID-19 panic may demonstrate heightened vigilance toward threat-related information, thereby encountering challenges in focusing attention and executing cognitive control strategies.

In contrast, TC practice has been shown to have beneficial effects on executive inhibitory control and brain activation patterns. TC is a mind–body practice that incorporates slow, deliberate movements, focused attention, and mindfulness techniques. Regular practice of TC has been associated with improvements in cognitive function, including executive inhibitory control. Several studies (Converse et al., 2020; Lv et al., 2022) have underscored the positive impact of TC on executive function in healthy college students. Additionally, Converse et al. (2020) and Douris et al. (2022) conducted a study using a repeated-measure crossover design with 16 college students and found the acute effects of 1-h sessions of martial arts had notable improvements in the post-test reaction time, particularly in the Stroop consistent and inconsistent conditions. The growing body of evidence supports the positive influence of TC on executive inhibitory control among the college student population, the requirement for balance and focus in TC exercises may enhance cognitive flexibility, facilitating faster and more accurate responses. Hence, the observed enhancement in inconsistent task performance likely stems from the positive modulation of attention and cognitive control through TC practice.

Functional neuroimaging studies have revealed alterations in brain activation patterns in individuals practicing TC, particularly in regions involved in attention, emotion regulation, and inhibitory control. These changes may reflect the adaptive neural plasticity induced by TC practice, leading to enhanced cognitive control and resilience to stress. An EEG biofeedback has demonstrated increased power in the four frequency bands of δ, θ, α, and β in the left frontal lobe of F3 after Yi Jin Jing Qigong intervention on early post-stroke depression, and the power of these bands in the right frontal lobe of F4 showed a decrease in the control group (Sun et al., 2022). It was similar to the results of our study, in which the α band power of F3 and F4 channels in the TC group increased compared with before the intervention, while the α band of the control group during the pandemic period significantly reduced compared to before the intervention. Another study of panic attack patients after EEG biofeedback treatment during the COVID-19 pandemic demonstrated increased α band amplitudes at C3 and C4 after 6 months of follow-up (Kopańska et al., 2022).

It is known that the prefrontal lobe is a key region for cognitive control and executive functions, and the increase in α band power may reflect TC’s modulation of prefrontal lobe function (Levit et al., 2020). α band are often associated with states of relaxation and rest but are also linked to focus and attention. These results align with previous findings indicating that participants in the 8-week training TC group exhibited significantly higher α1 and α2 power compared to the control group, suggesting increased focus and attention in a pilot study using resting EEG signals (Zhang et al., 2022; Wang and Ali, 2023; Zhang et al., 2023). The practice of TC, requiring participants to stay focused during movements and on breathing control, may contribute to the observed increase inα band. In the prefrontal region, increased α band may reflect a relaxed yet focused state, helping balance the allocation of cognitive resources and increase cognitive flexibility, thereby improving task performance. Similarly, θ band, often associated with relaxation and cognitive integration, increased in prefrontal regions in the TC group, reflecting a state of relaxation induced by TC practice. Enhancements in this state may facilitate better processing of complex cognitive tasks and promote cognitive integration in prefrontal regions.

Surprisingly, we also found there were interaction effect of group and time in both executive inhibitory control performance and frontal lobe activation. It emphasized that TC potentially influenced the brainwave activity of the frontal lobe region through various pathways. Firstly, TC practice emphasized the integration of body and mind, inducing a meditative and relaxed state through smooth movements and controlled breathing. This state might alleviate anxiety, reduce psychological stress, and, consequently, enhance cognitive performance in the frontal lobe. Secondly, TC’s movement requirements for focus and coordination heightened participants’ attention levels, aiding in coping with cognitive conflicts. Lastly, TC might directly impact frontal lobe EEG wave activity by balancing the autonomic nervous system, subsequently influencing cognitive function.

COVID-19 pandemic may negatively impact executive inhibitory control and brain activation results through increased stress and anxiety, whereas TC practice may offer a potential intervention to mitigate these effects and enhance cognitive resilience. TC, often hailed as “moving meditation,” seamlessly blends gentle body movements with mindfulness and deep breathing techniques to enhance both physical and mental well-being while fostering cultural appreciation and social cohesion. By encouraging mindfulness and cultivating a present-focused awareness, TC effectively mitigates stress, anxiety, and depression while inducing a sense of relaxation and tranquility. Its practice fosters mental clarity and emotional equilibrium and synergizes with existing meditation routines to amplify mindfulness and relaxation benefits. Moreover, TC’s rich historical, philosophical, and cultural tapestry serves to deepen understanding and appreciation of diverse traditions, fostering cross-cultural empathy and acceptance. Simultaneously, group TC sessions facilitate meaningful social interactions and community engagement, further enriching the holistic experience. Nevertheless, it is crucial to acknowledge the limitations of this study, although TC is a low-intensity exercise, it still causes a certain amount of fatigue in the body. Secondly, for those students who are socially anxious or shy, there may be a certain amount of social pressure. Finally, TC requires a certain amount of time investment and practice, which may conflict with students’ academic pressures and be distracting or distracting, especially for tasks that require concentrated study. These may affect their cognitive executive functions such as attention, working memory, and problem-solving. In addition, potential generalizability issues under specific experimental conditions. Further long-term research and more comprehensive EEG analyses may contribute to confirming these findings and deepening our understanding of the intricate impact mechanisms of TC on cognition and neural function.

## 5 Conclusion

The TC group’s heightened accuracy, shortened reaction times in inconsistent tasks, and the overall increase in frontal lobe α, β, and θ wave power offer preliminary evidence for the positive modulation of frontal lobe activity by TC. This finding not only bolsters the cognitive merits of TC as a mind–body practice but also provides avenues for exploring the neural mechanisms of TC. However, it remains imperative to validate these conclusions in larger samples and diverse experimental conditions to ensure the reliability and generalizability of the results.

## Data availability statement

The original contributions presented in the study are included in the article/supplementary material, further inquiries can be directed to the corresponding author.

## Ethics statement

This study protocol was approved by the Center for Ethics in Human Research, Khon Kaen University, and Recorded No.4.2.01: 6/2565 (Reference No. HE652012). All participants provided written informed consent before enrolling in the study.

## Author contributions

MW: Conceptualization, Data curation, Formal analysis, Investigation, Methodology, Project administration, Resources, Software, Validation, Visualization, Writing – original draft, Writing – review & editing. BL: Conceptualization, Data curation, Formal analysis, Funding acquisition, Investigation, Methodology, Project administration, Resources, Software, Supervision, Validation, Visualization, Writing – original draft, Writing – review & editing.
